# Supplementary material for: Colorectal Surgery in the COVID-19 Era: A Systematic Review and Meta-Analysis
Source: Cancers (Basel). 2022 Feb 27;14(5):1229. doi: 10.3390/cancers14051229 (PMC8909364; doi:10.3390/cancers14051229)
Supplement: Supplementary file 1 [file cancers-14-01229-s001.zip › cancers-1549321-supplementary.pdf]

cancers

MDPI

Supplementary Material

Colorectal Surgery in the COVID-19 Era: A Systematic Review and Meta-Analysis

Nikolaos Pararas, Anastasia Pikouli, Dimitrios Papaconstantinou, Georgios Bagias, Constantinos Nastos, Andreas Pikoulis, Dionysios Dellaportas, Panagis Lykoudis and Emmanouil Pikoulis

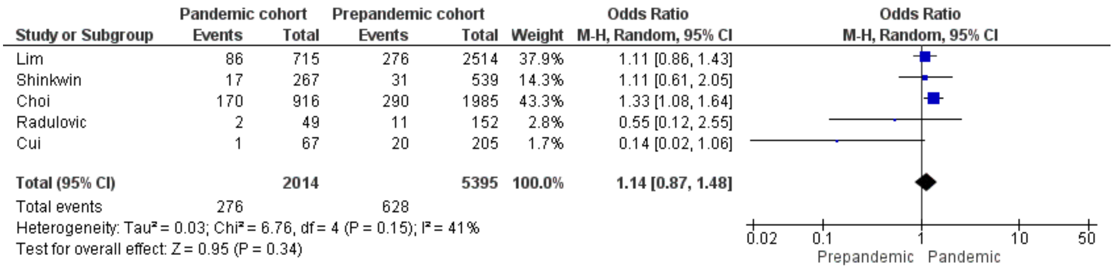

Study or Subgroup	Pandemic cohort Events	Pandemic cohort Total	Prepandemic cohort Events	Prepandemic cohort Total	Weight	Odds Ratio M-H, Random, 95% CI
Lim	86	715	276	2514	37.9%	1.11 [0.86, 1.43]
Shinkwin	17	267	31	539	14.3%	1.11 [0.61, 2.05]
Choi	170	916	290	1985	43.3%	1.33 [1.08, 1.64]
Radulovic	2	49	11	152	2.8%	0.55 [0.12, 2.55]
Cui	1	67	20	205	1.7%	0.14 [0.02, 1.06]
**Total (95% CI)**		**2014**		**5395**	**100.0%**	**1.14 [0.87, 1.48]**
Total events	276		628			

Heterogeneity: Tau² = 0.03; Chi² = 6.76, df = 4 (P = 0.15); I² = 41%
Test for overall effect: Z = 0.95 (P = 0.34)

Figure S1. Tis-T1 stage forest plot.

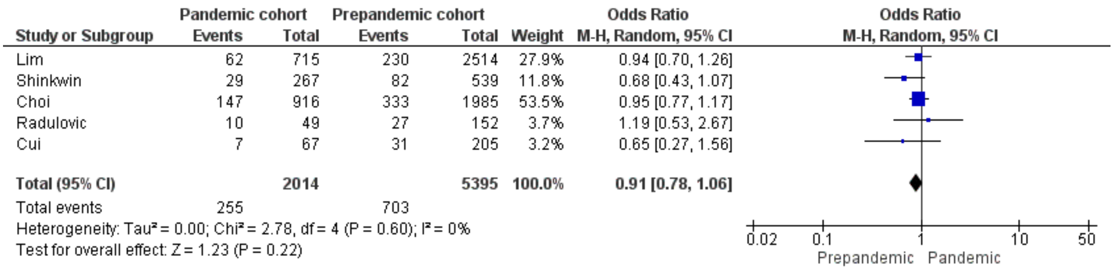

Study or Subgroup	Pandemic cohort Events	Pandemic cohort Total	Prepandemic cohort Events	Prepandemic cohort Total	Weight	Odds Ratio M-H, Random, 95% CI
Lim	62	715	230	2514	27.9%	0.94 [0.70, 1.26]
Shinkwin	29	267	82	539	11.8%	0.68 [0.43, 1.07]
Choi	147	916	333	1985	53.5%	0.95 [0.77, 1.17]
Radulovic	10	49	27	152	3.7%	1.19 [0.53, 2.67]
Cui	7	67	31	205	3.2%	0.65 [0.27, 1.56]
**Total (95% CI)**		**2014**		**5395**	**100.0%**	**0.91 [0.78, 1.06]**
Total events	255		703			

Heterogeneity: Tau² = 0.00; Chi² = 2.78, df = 4 (P = 0.60); I² = 0%
Test for overall effect: Z = 1.23 (P = 0.22)

Figure S2. T2 stage forest plot.

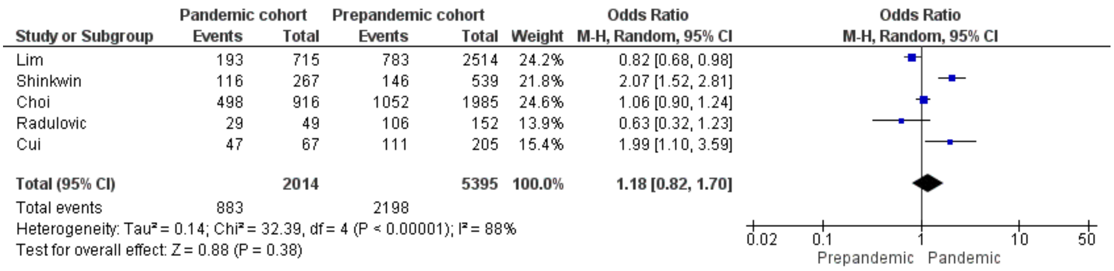

Study or Subgroup	Pandemic cohort Events	Pandemic cohort Total	Prepandemic cohort Events	Prepandemic cohort Total	Weight	Odds Ratio M-H, Random, 95% CI
Lim	193	715	783	2514	24.2%	0.82 [0.68, 0.98]
Shinkwin	116	267	146	539	21.8%	2.07 [1.52, 2.81]
Choi	498	916	1052	1985	24.6%	1.06 [0.90, 1.24]
Radulovic	29	49	106	152	13.9%	0.63 [0.32, 1.23]
Cui	47	67	111	205	15.4%	1.99 [1.10, 3.59]
**Total (95% CI)**		**2014**		**5395**	**100.0%**	**1.18 [0.82, 1.70]**
Total events	883		2198			

Heterogeneity: Tau² = 0.14; Chi² = 32.39, df = 4 (P < 0.00001); I² = 88%
Test for overall effect: Z = 0.88 (P = 0.38)

Figure S3. T3 stage forest plot.

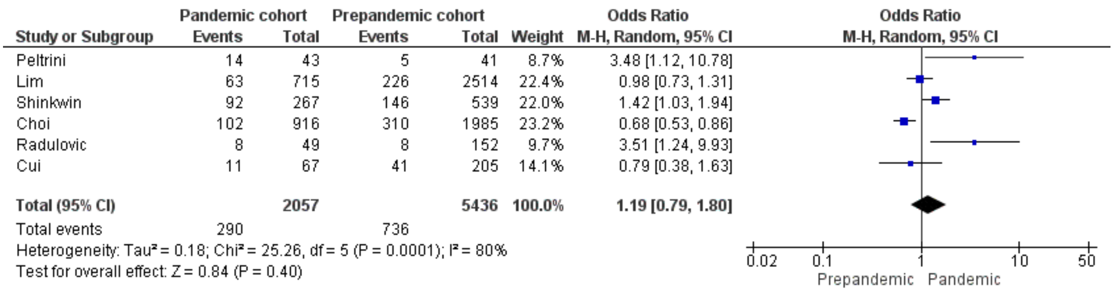

Study or Subgroup	Pandemic cohort Events	Pandemic cohort Total	Prepandemic cohort Events	Prepandemic cohort Total	Weight	Odds Ratio M-H, Random, 95% CI
Peltrini	14	43	5	41	8.7%	3.48 [1.12, 10.78]
Lim	63	715	226	2514	22.4%	0.98 [0.73, 1.31]
Shinkwin	92	267	146	539	22.0%	1.42 [1.03, 1.94]
Choi	102	916	310	1985	23.2%	0.68 [0.53, 0.86]
Radulovic	8	49	8	152	9.7%	3.51 [1.24, 9.93]
Cui	11	67	41	205	14.1%	0.79 [0.38, 1.63]
**Total (95% CI)**		**2057**		**5436**	**100.0%**	**1.19 [0.79, 1.80]**
Total events	290		736			

Heterogeneity: Tau² = 0.18; Chi² = 25.26, df = 5 (P = 0.0001); I² = 80%
Test for overall effect: Z = 0.84 (P = 0.40)

Figure S4. T4 stage forest plot.

	Pandemic cohort		Prepandemic cohort			Odds Ratio
**Study or Subgroup**	**Events**	**Total**	**Events**	**Total**	**Weight**	**M-H, Random, 95% CI**
Peltrini	20	43	13	41	1.5%	1.87 [0.77, 4.56]
Lim	168	715	596	2514	31.8%	0.99 [0.81, 1.20]
Shinkwin	152	267	292	539	14.0%	1.12 [0.83, 1.50]
Choi	328	916	734	1985	46.0%	0.95 [0.81, 1.12]
Radulovic	27	49	74	152	2.9%	1.29 [0.68, 2.47]
Cui	25	67	88	205	3.8%	0.79 [0.45, 1.40]
**Total (95% CI)**		**2057**		**5436**	**100.0%**	**1.00 [0.89, 1.11]**
Total events	720		1797			

Heterogeneity: Tau² = 0.00; Chi² = 4.10, df = 5 (P = 0.54); I² = 0%
Test for overall effect: Z = 0.05 (P = 0.96)

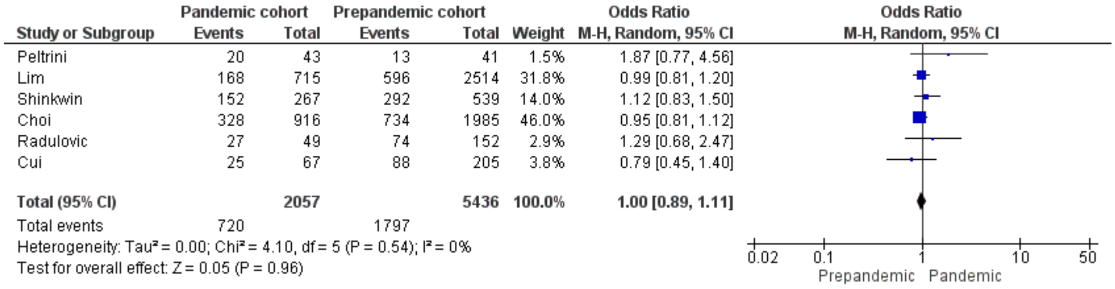

Figure S5. Node positive tumors forest plot.

	Pandemic cohort		Prepandemic cohort			Odds Ratio
**Study or Subgroup**	**Events**	**Total**	**Events**	**Total**	**Weight**	**M-H, Random, 95% CI**
Peltrini	11	43	6	41	10.4%	2.01 [0.66, 6.05]
Lim	134	715	419	2514	22.2%	1.15 [0.93, 1.43]
Shinkwin	152	267	145	539	21.2%	3.59 [2.64, 4.89]
Kuryba	401	3227	1425	11703	22.9%	1.02 [0.91, 1.15]
Radulovic	7	49	12	152	11.7%	1.94 [0.72, 5.25]
Cui	6	67	13	205	11.5%	1.45 [0.53, 3.99]
**Total (95% CI)**		**4368**		**15154**	**100.0%**	**1.65 [1.02, 2.67]**
Total events	711		2020			

Heterogeneity: Tau² = 0.26; Chi² = 57.58, df = 5 (P < 0.00001); I² = 91%
Test for overall effect: Z = 2.05 (P = 0.04)

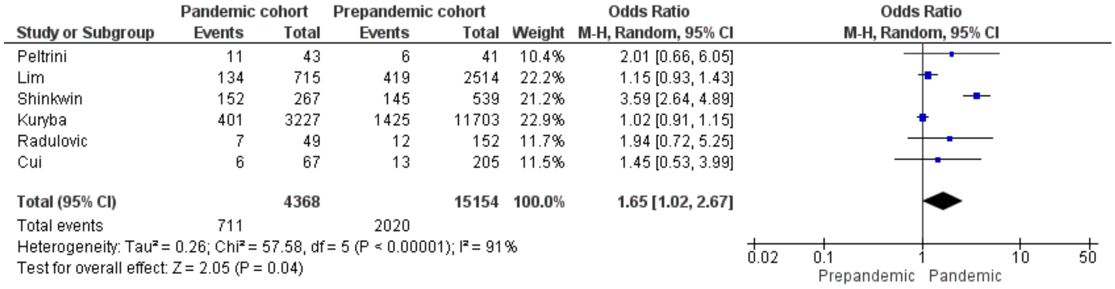

Figure S6. Metastatic tumors forest plot.

	Pandemic cohort		Prepandemic cohort			Odds Ratio
**Study or Subgroup**	**Events**	**Total**	**Events**	**Total**	**Weight**	**M-H, Random, 95% CI**
Donlon	347	1631	506	1093	16.8%	0.31 [0.27, 0.37]
Peltrini	13	43	17	41	11.6%	0.61 [0.25, 1.50]
Kuryba	1270	3227	4496	11703	17.0%	1.04 [0.96, 1.13]
Radulovic	13	49	35	152	13.0%	1.21 [0.58, 2.53]
Xu	166	710	181	828	16.5%	1.09 [0.86, 1.39]
Cui	12	67	23	205	12.8%	1.73 [0.81, 3.69]
Mizuno	13	31	36	92	12.2%	1.12 [0.49, 2.57]
**Total (95% CI)**		**5758**		**14114**	**100.0%**	**0.88 [0.51, 1.52]**
Total events	1834		5294			

Heterogeneity: Tau² = 0.45; Chi² = 169.33, df = 6 (P < 0.00001); I² = 96%
Test for overall effect: Z = 0.44 (P = 0.66)

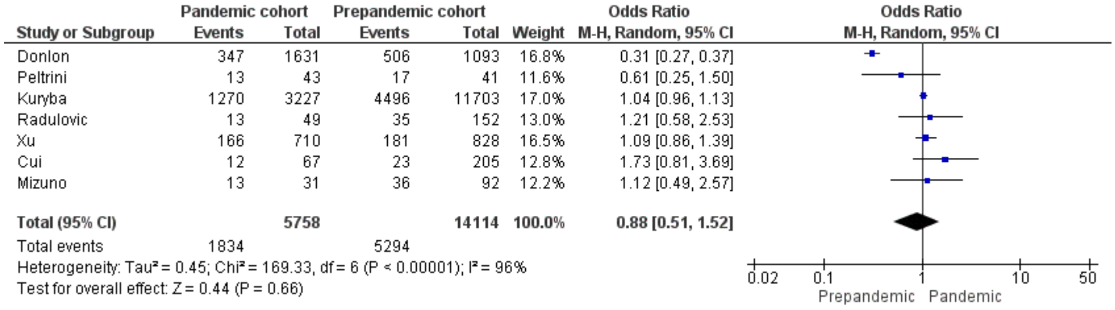

Figure S7. Right-sided tumors forest plot.

	Pandemic cohort		Prepandemic cohort			Odds Ratio
**Study or Subgroup**	**Events**	**Total**	**Events**	**Total**	**Weight**	**M-H, Random, 95% CI**
Donlon	385	1631	525	1093	17.2%	0.33 [0.28, 0.39]
Peltrini	19	43	12	41	11.1%	1.91 [0.78, 4.72]
Kuryba	1093	3227	4012	11703	17.4%	0.98 [0.90, 1.07]
Radulovic	18	49	57	152	13.3%	0.97 [0.50, 1.89]
Xu	211	710	276	828	17.0%	0.85 [0.68, 1.05]
Cui	20	67	30	205	13.4%	2.48 [1.29, 4.76]
Mizuno	7	31	31	92	10.7%	0.57 [0.22, 1.48]
**Total (95% CI)**		**5758**		**14114**	**100.0%**	**0.91 [0.56, 1.50]**
Total events	1753		4943			

Heterogeneity: Tau² = 0.36; Chi² = 147.64, df = 6 (P < 0.00001); I² = 96%
Test for overall effect: Z = 0.36 (P = 0.72)

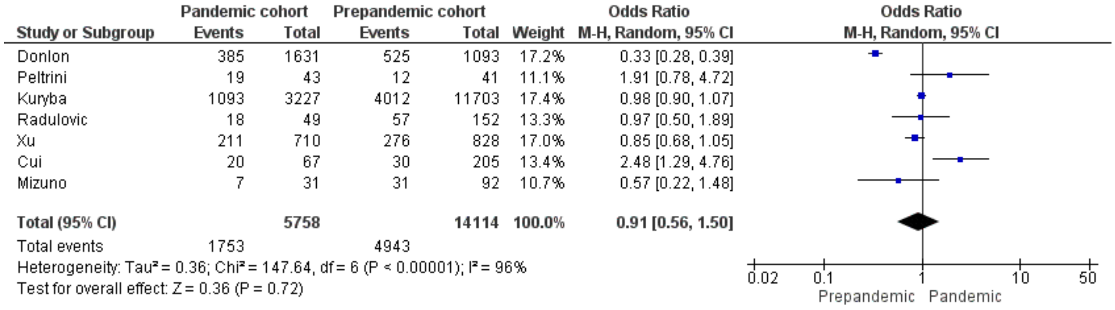

Figure S8. Left-sided tumors forest plot.

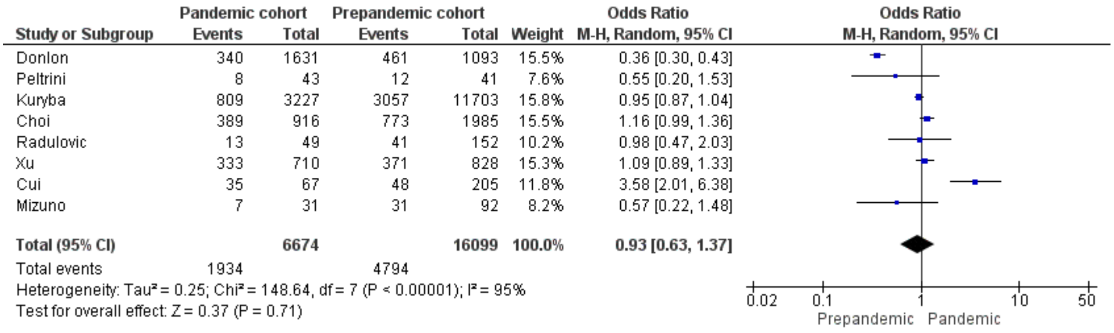

Figure S9. Rectal tumors forest plot.

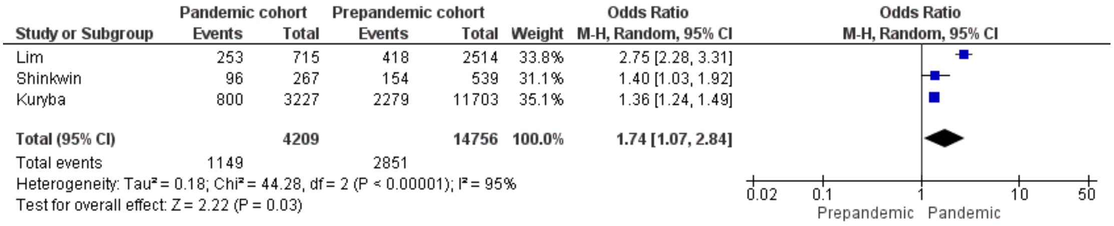

Figure S10. Emergency presentation forest plot.

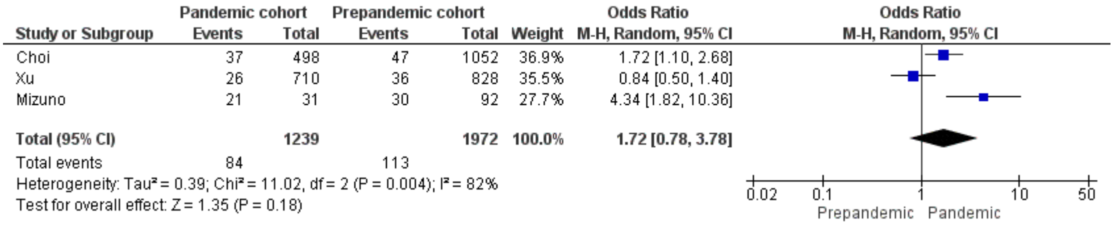

Figure S11. Complicated tumors forest plot.

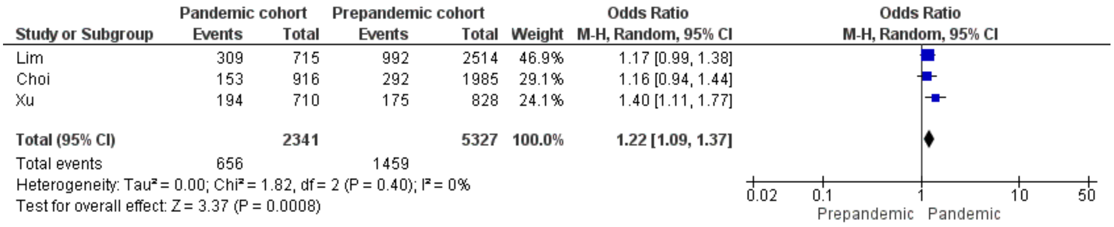

Figure S12. Neoadjuvant utilization forest plot.

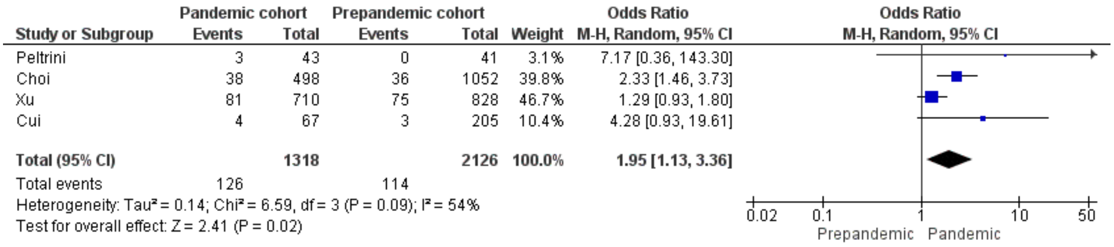

Figure S13. Palliative intent surgery forest plot.

Study or Subgroup	Prepandemic cohort Events	Total	Pandemic cohort Events	Total	Weight	Odds Ratio M-H, Random, 95% CI
Donlon	114	1093	34	1631	16.8%	5.47 [3.70, 8.09]
Kuryba	5749	11703	865	3227	18.0%	2.64 [2.42, 2.87]
Choi	1314	1985	566	916	17.9%	1.21 [1.03, 1.42]
Radulovic	36	152	13	49	14.2%	0.86 [0.41, 1.79]
Xu	327	828	351	710	17.7%	0.67 [0.55, 0.82]
Cui	140	205	47	67	15.3%	0.92 [0.50, 1.67]
**Total (95% CI)**		**15966**		**6600**	**100.0%**	**1.47 [0.81, 2.69]**
Total events	7680		1876			

Heterogeneity: Tau² = 0.52; Chi² = 225.59, df = 5 (P < 0.00001); I² = 98%
Test for overall effect: Z = 1.27 (P = 0.20)

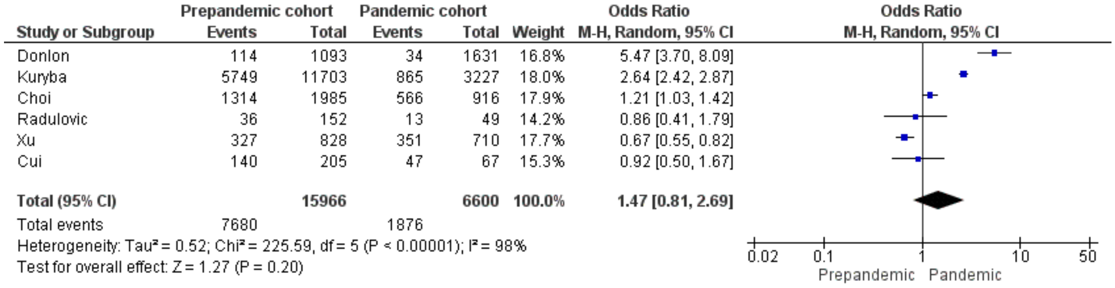

Figure S14. Minimally invasive surgery forest plot.

Study or Subgroup	Prepandemic cohort Events	Total	Pandemic cohort Events	Total	Weight	Odds Ratio M-H, Random, 95% CI
Donlon	118	1093	52	1631	21.3%	3.67 [2.63, 5.14]
Kuryba	1134	11703	275	3227	22.7%	1.15 [1.00, 1.32]
Radulovic	33	152	13	49	16.7%	0.77 [0.37, 1.61]
Xu	109	828	123	710	21.8%	0.72 [0.55, 0.96]
Cui	31	205	16	67	17.5%	0.57 [0.29, 1.12]
**Total (95% CI)**		**13981**		**5684**	**100.0%**	**1.10 [0.62, 1.96]**
Total events	1425		479			

Heterogeneity: Tau² = 0.38; Chi² = 61.59, df = 4 (P < 0.00001); I² = 94%
Test for overall effect: Z = 0.33 (P = 0.74)

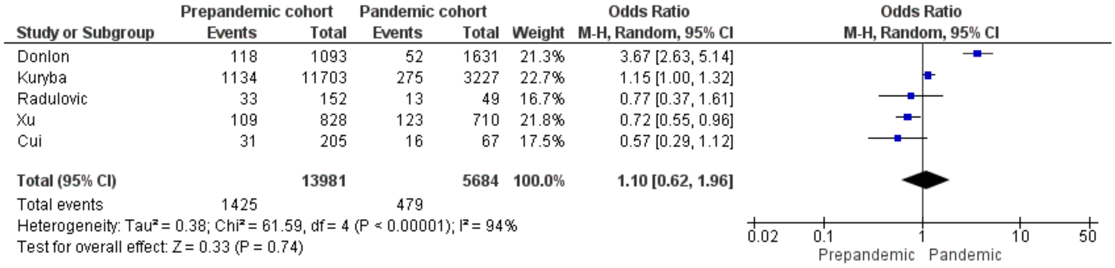

Figure S15. Stoma formation forest plot.

Study or Subgroup	Pandemic cohort Events	Total	Prepandemic cohort Events	Total	Weight	Odds Ratio M-H, Random, 95% CI
Xu	28	710	42	828	68.3%	0.77 [0.47, 1.25]
Cui	9	67	21	205	31.7%	1.36 [0.59, 3.13]
**Total (95% CI)**		**777**		**1033**	**100.0%**	**0.92 [0.55, 1.55]**
Total events	37		63			

Heterogeneity: Tau² = 0.04; Chi² = 1.34, df = 1 (P = 0.25); I² = 25%
Test for overall effect: Z = 0.31 (P = 0.76)

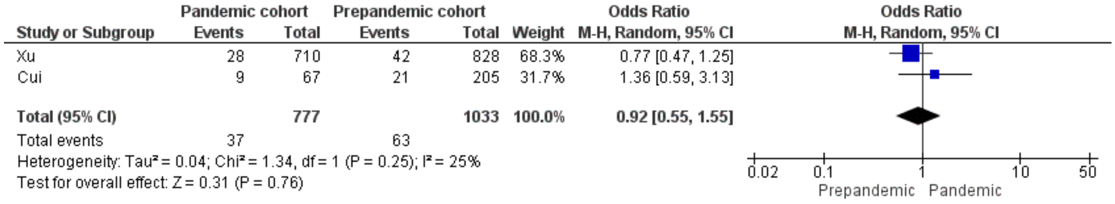

Figure S16. Morbidity forest plot.

Study or Subgroup	Pandemic cohort Mean	SD	Total	Prepandemic cohort Mean	SD	Total	Weight	Mean Difference IV, Random, 95% CI
Radulovic	10.77	6.09	49	9.58	3.64	152	25.9%	1.19 [-0.61, 2.99]
Xu	8.4	3.1	710	7.2	2.8	828	42.7%	1.20 [0.90, 1.50]
Cui	9.6	3.7	67	10.6	7.5	205	31.4%	-1.00 [-2.36, 0.36]
**Total (95% CI)**			**826**			**1185**	**100.0%**	**0.51 [-0.93, 1.94]**

Heterogeneity: Tau² = 1.24; Chi² = 9.66, df = 2 (P = 0.008); I² = 79%
Test for overall effect: Z = 0.69 (P = 0.49)

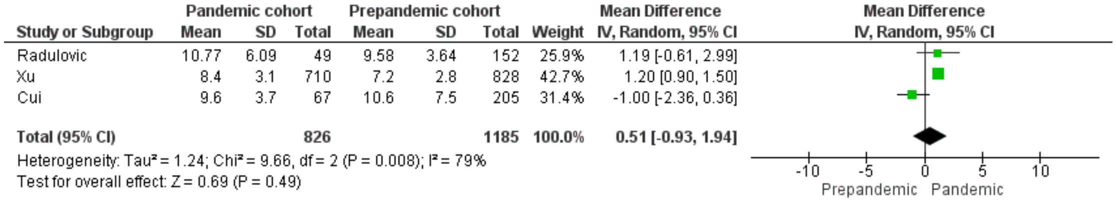

Figure S17. Length of hospital stay forest plot.

Study or Subgroup	Pandemic cohort Mean	SD	Total	Prepandemic cohort Mean	SD	Total	Weight	Mean Difference IV, Random, 95% CI
Peltrini	29.5	18.8	43	20.6	14.5	41	17.1%	8.90 [1.74, 16.06]
Radulovic	17.93	10.16	49	18.32	9.23	152	38.3%	-0.39 [-3.59, 2.81]
Cui	19.37	7.86	67	18.93	10.48	205	44.6%	0.44 [-1.93, 2.81]
**Total (95% CI)**			**159**			**398**	**100.0%**	**1.57 [-1.99, 5.13]**

Heterogeneity: Tau² = 5.95; Chi² = 5.53, df = 2 (P = 0.06); I² = 64%
Test for overall effect: Z = 0.86 (P = 0.39)

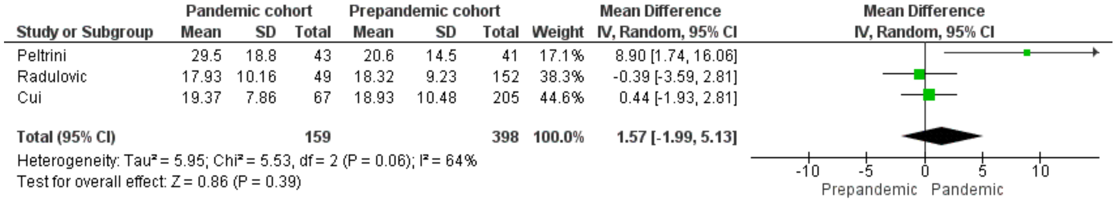

Figure S18. Lymph node harvest forest plot.

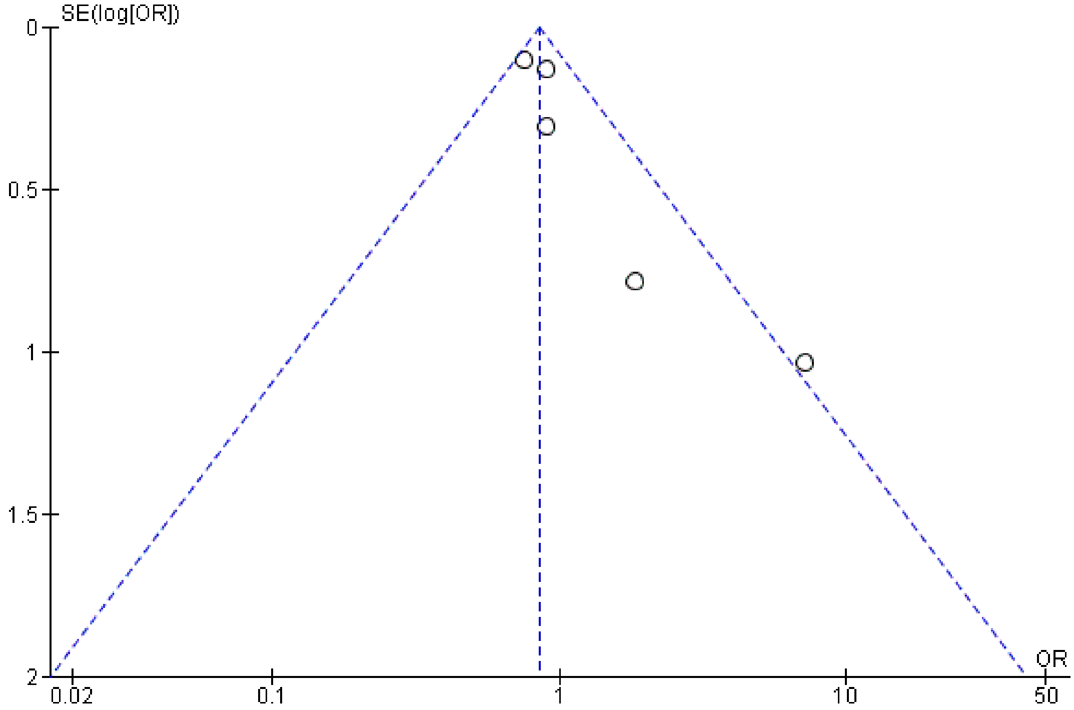

Figure S19. Tis-T1 stage funnel plot.

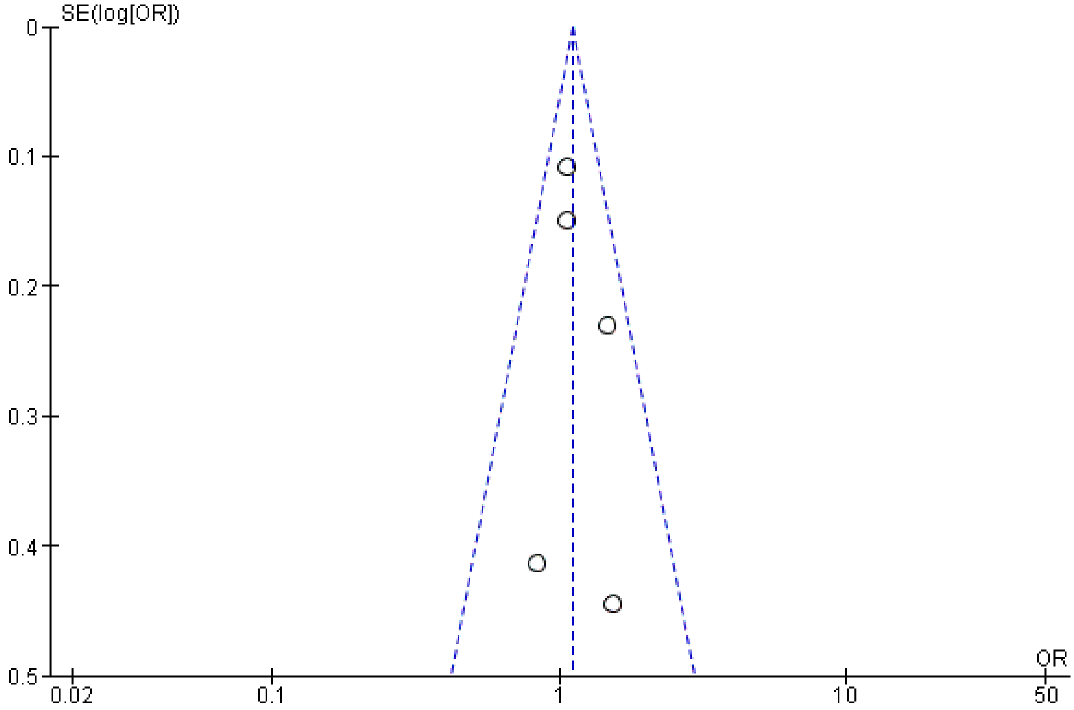

Figure S20. T2 stage funnel plot.

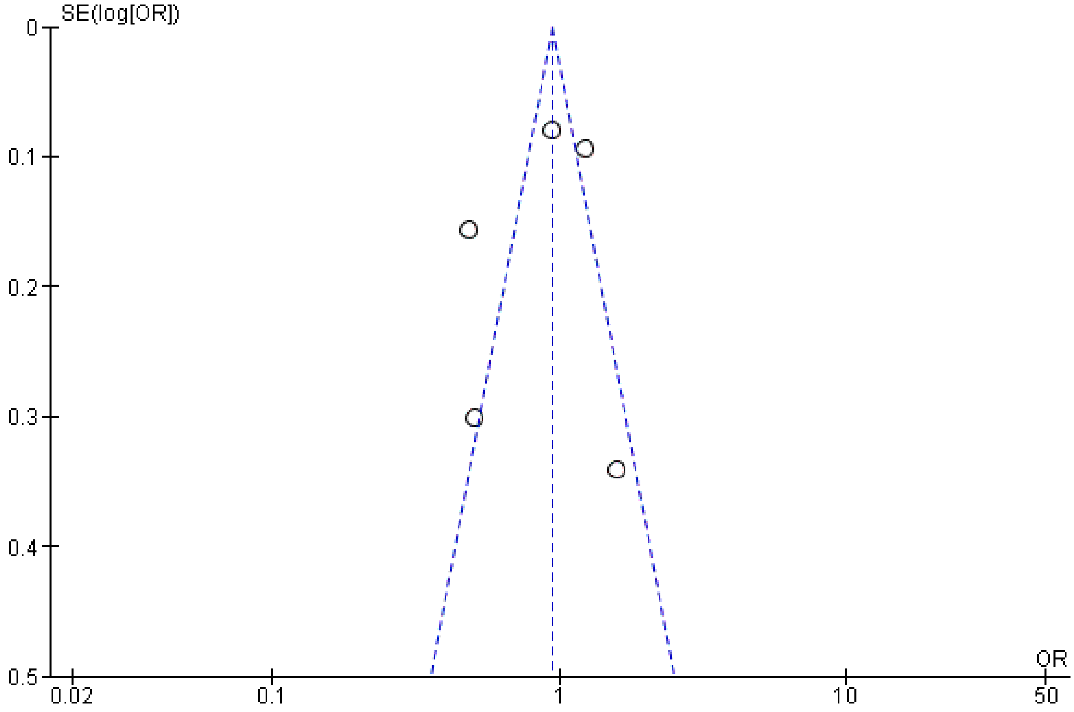

Figure S21. T3 stage funnel plot.

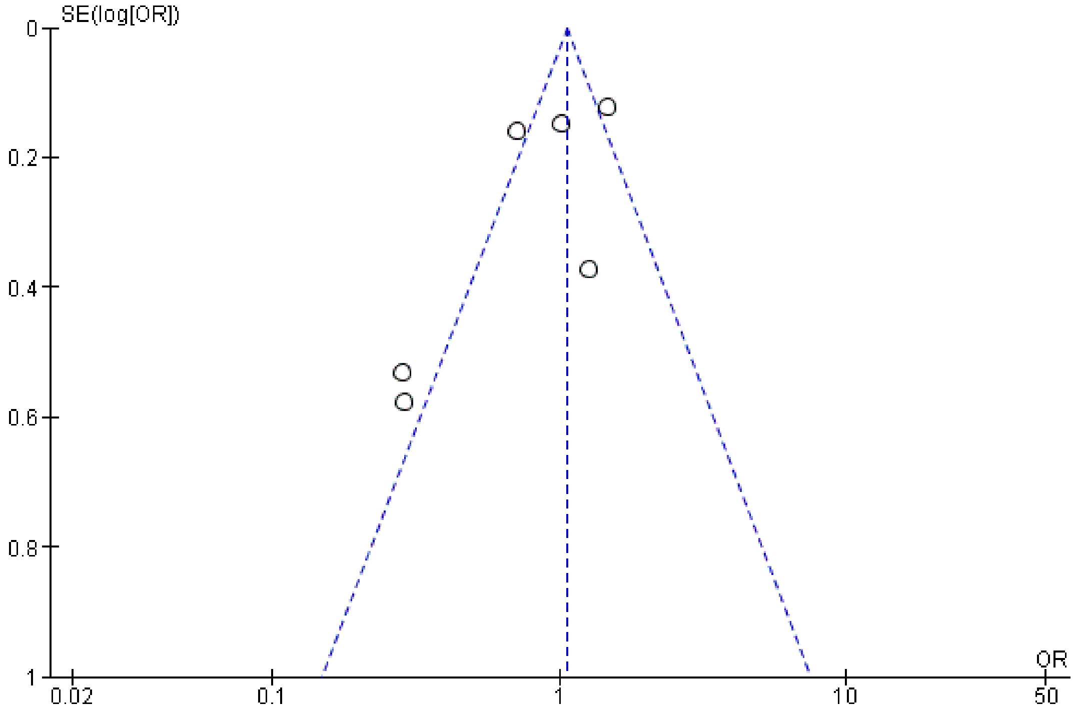

Figure S22. T4 stage funnel plot.

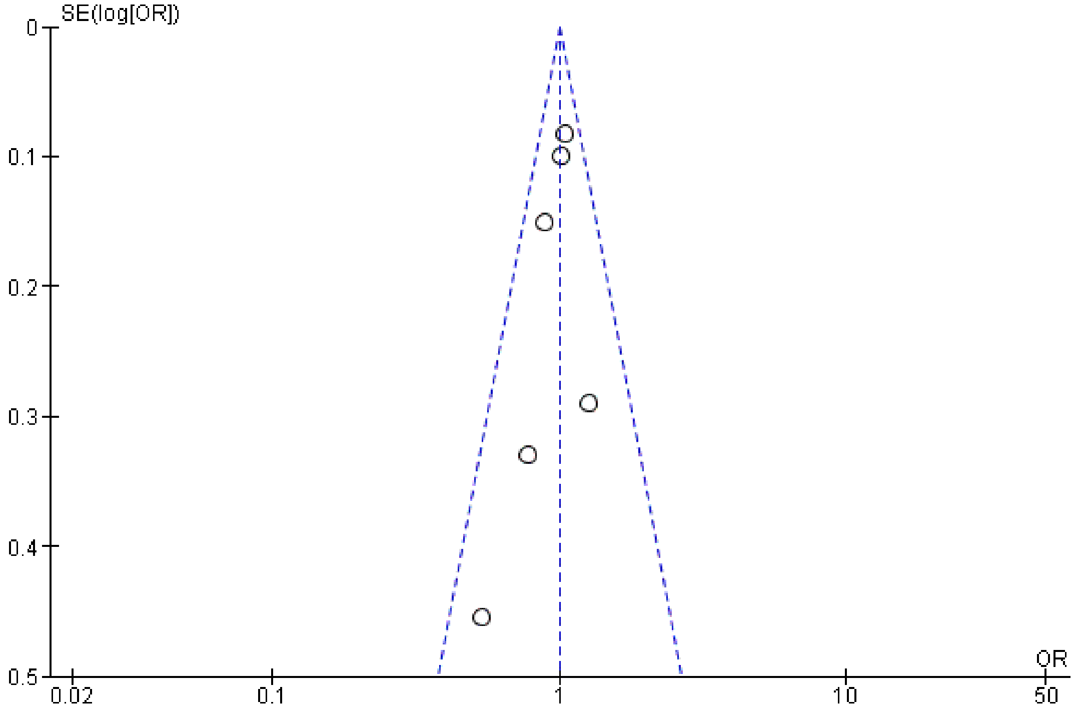

Figure S23. Node positive tumors funnel plot.

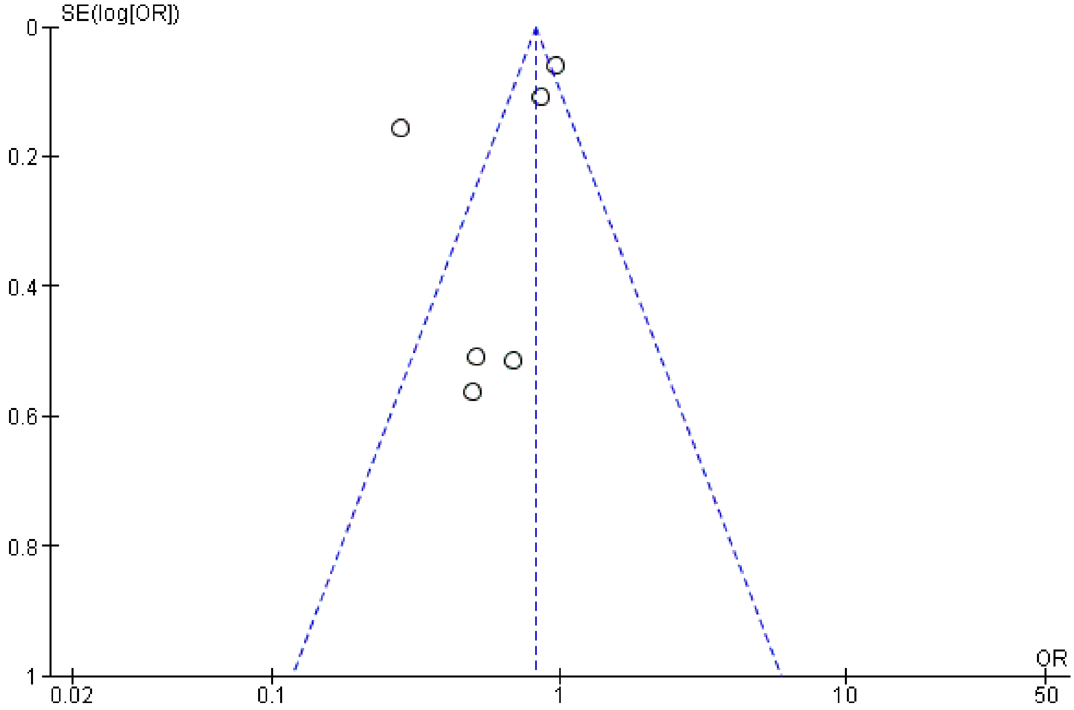

Figure S24. Metastatic tumors funnel plot.

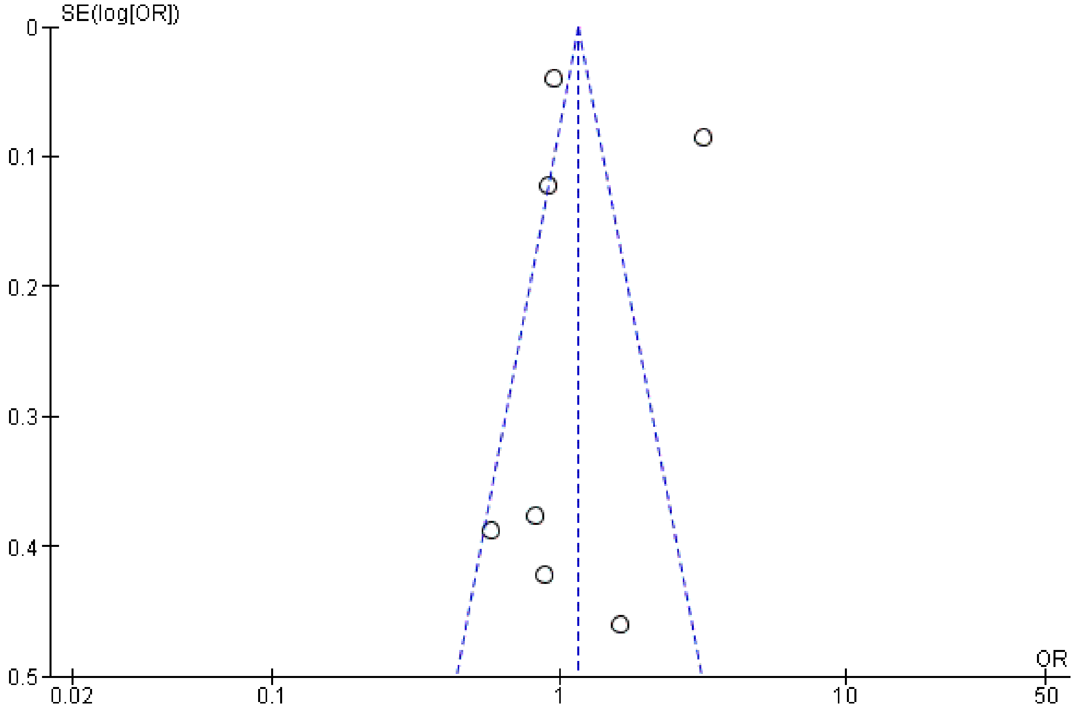

Figure S25. Right-sided tumors funnel plot.

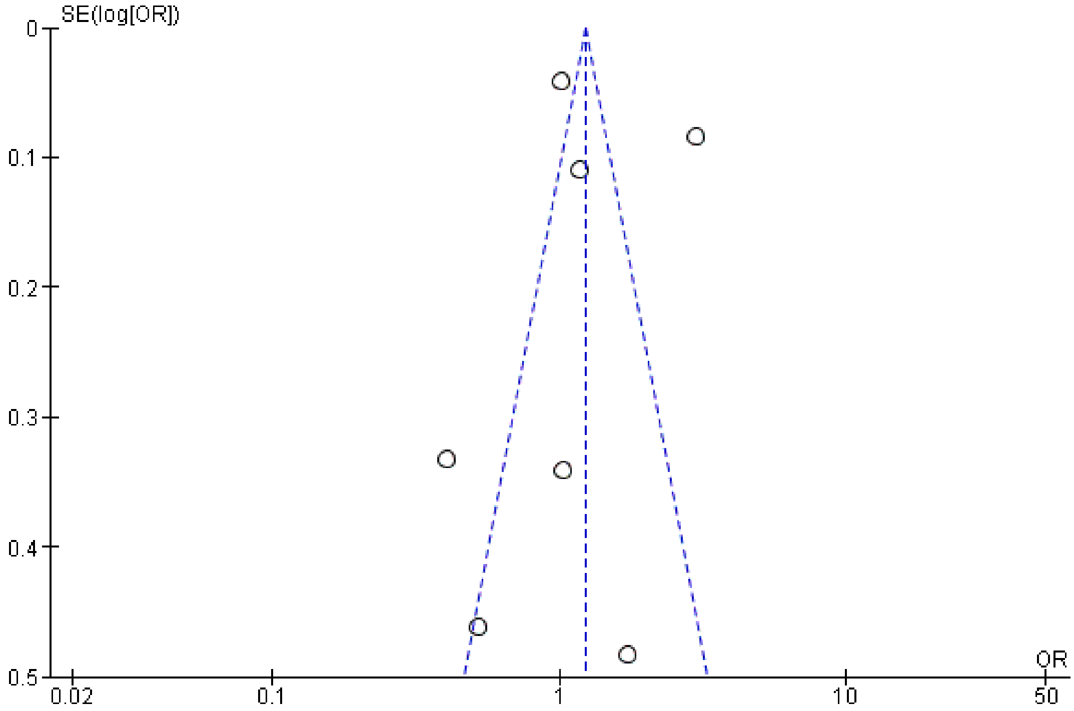

Figure S26. Left-sided tumors funnel plot.

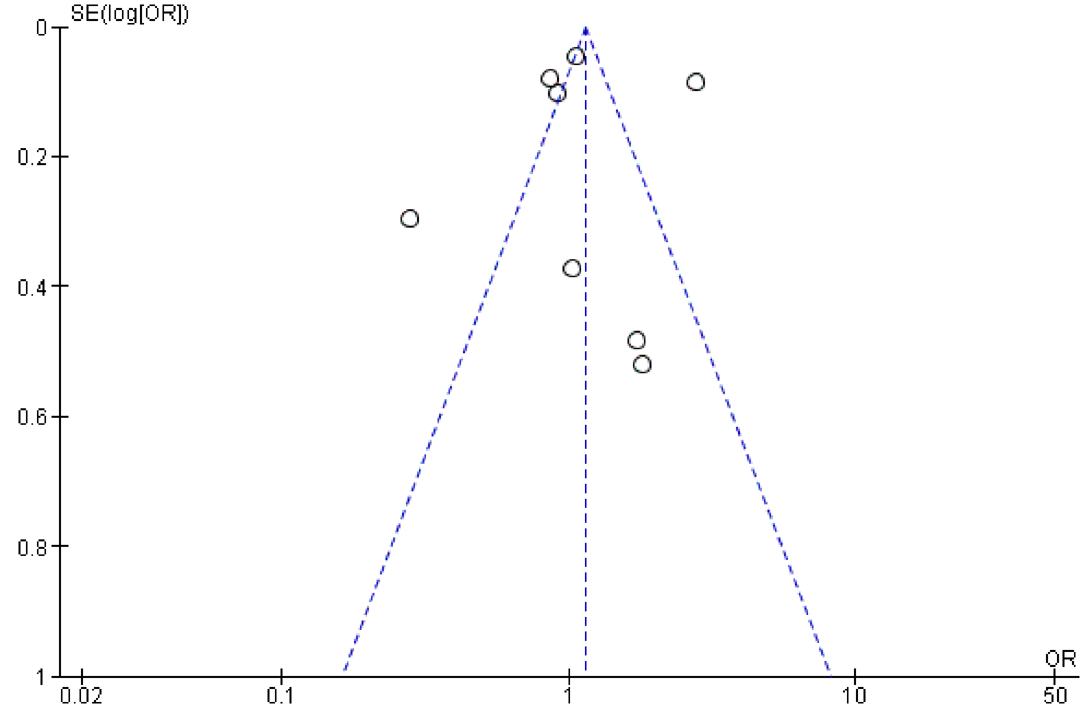

Figure S27. Rectal tumors funnel plot.

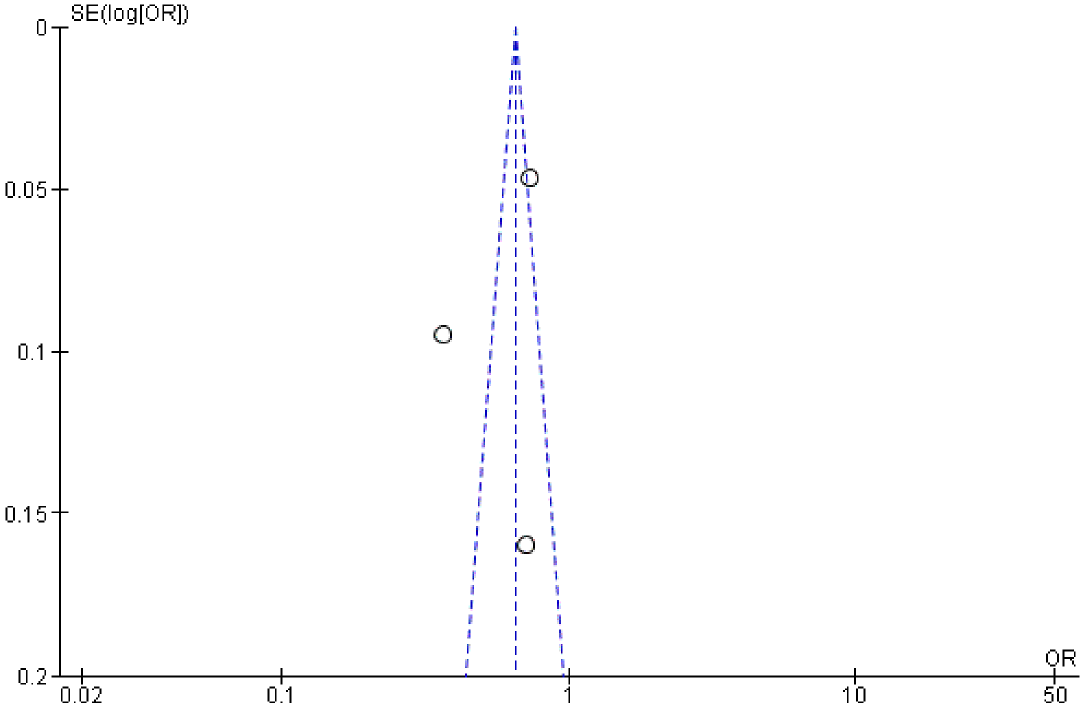

Figure S28. Emergency presentation funnel plot.

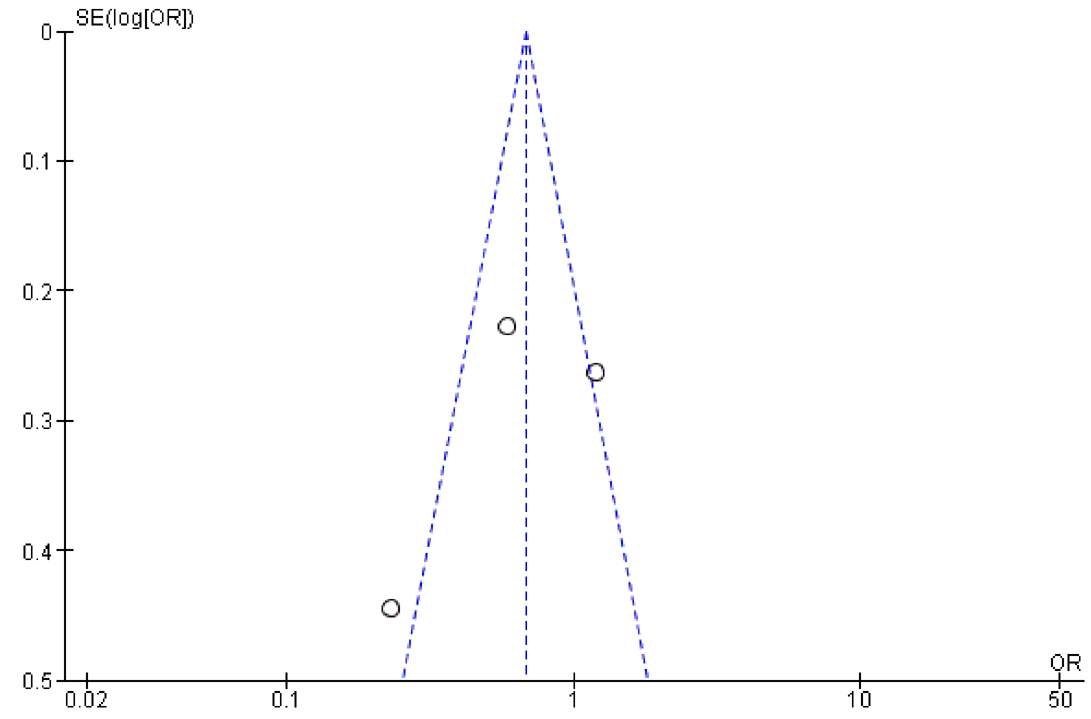

Figure S29. Funnel plot of complicated tumors.

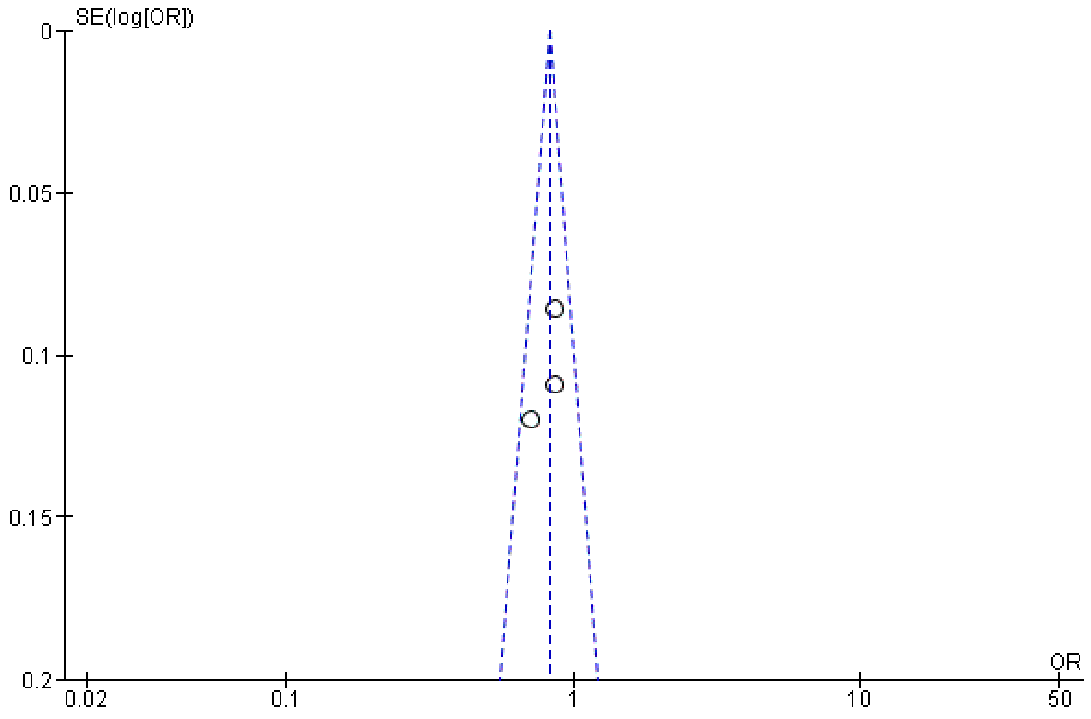

Figure S30. Neoadjuvant utilization funnel plot.

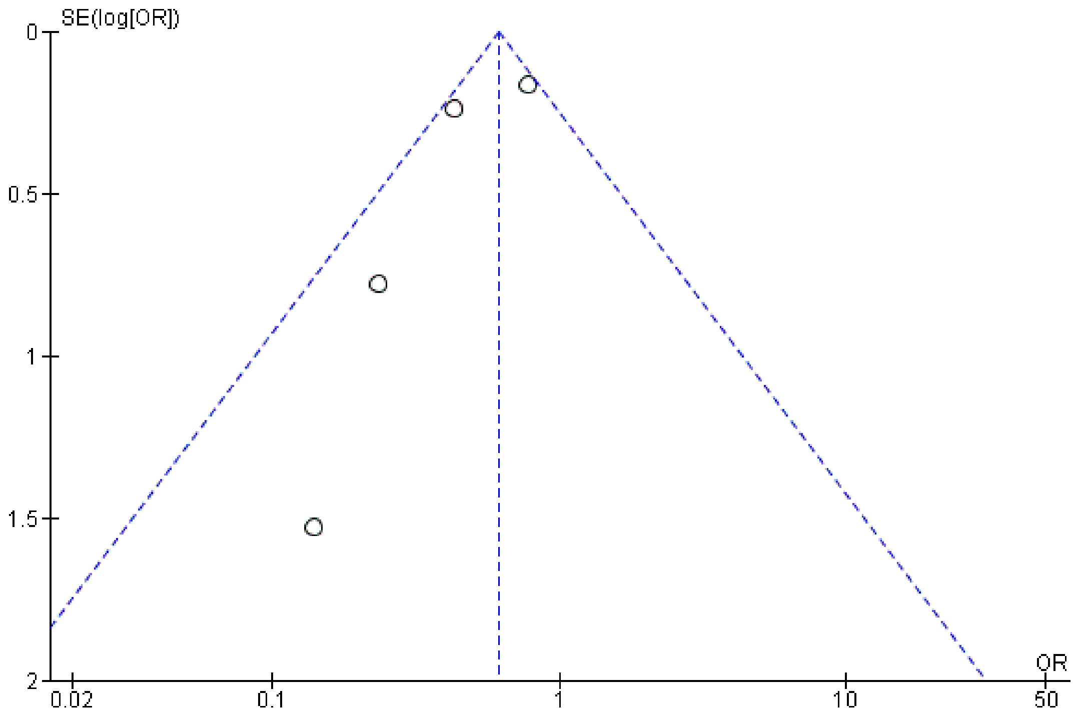

Figure S31. Palliative intent surgery funnel plot.

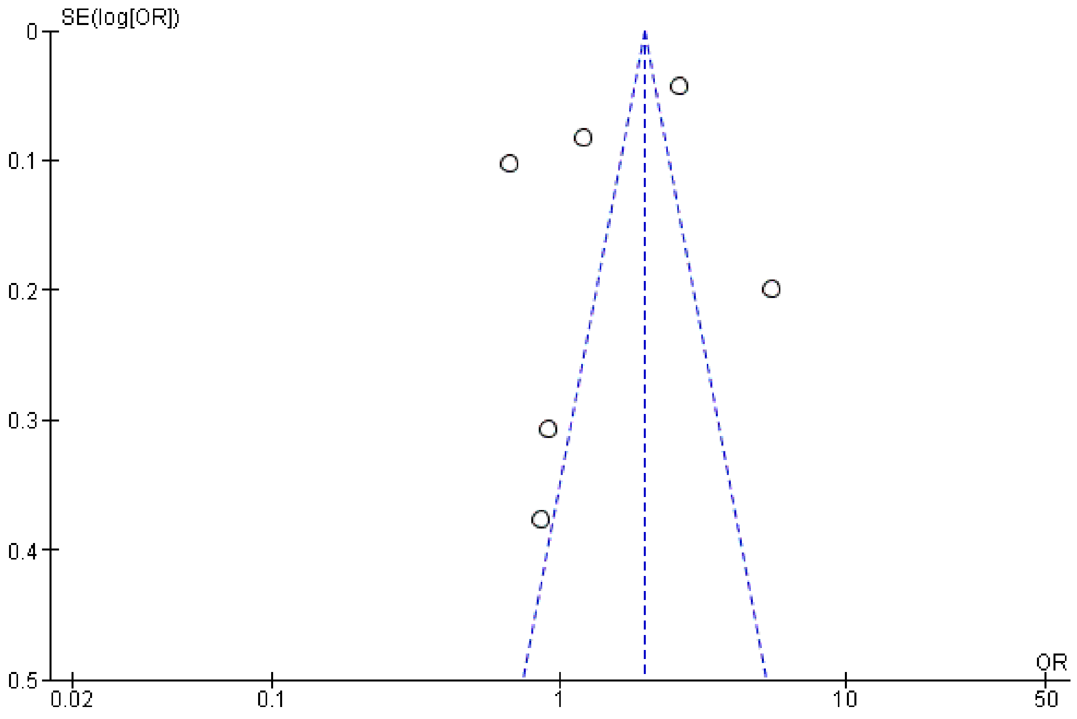

Figure S32. Minimally invasive surgery funnel plot.

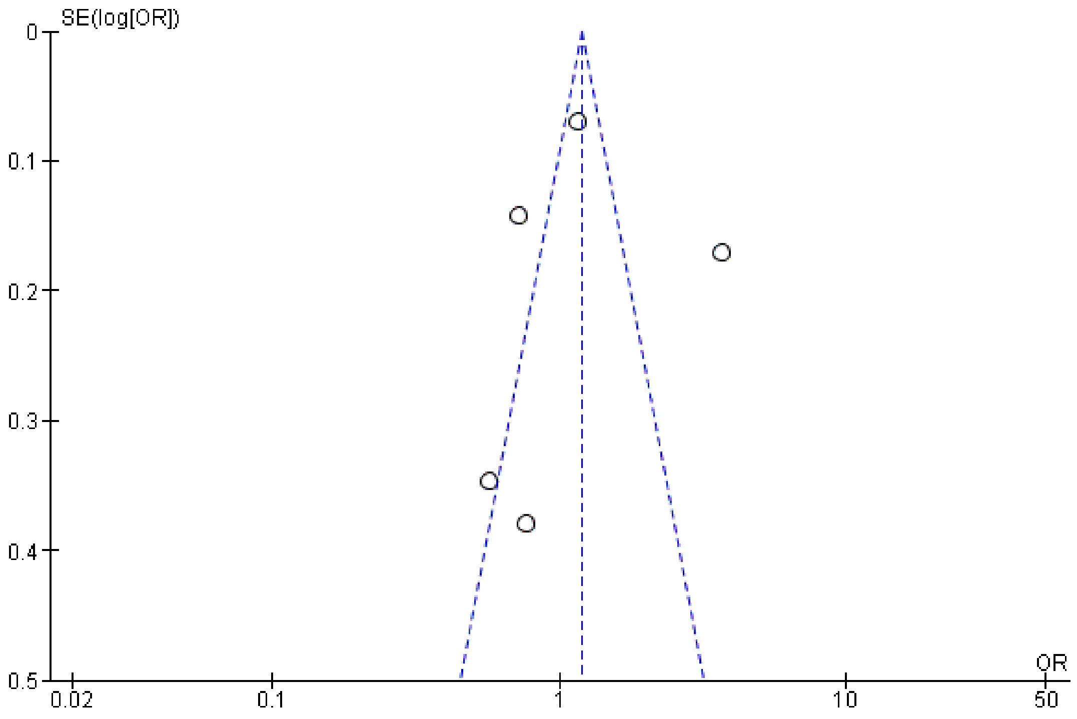

Figure S33. Stoma formation funnel plot.

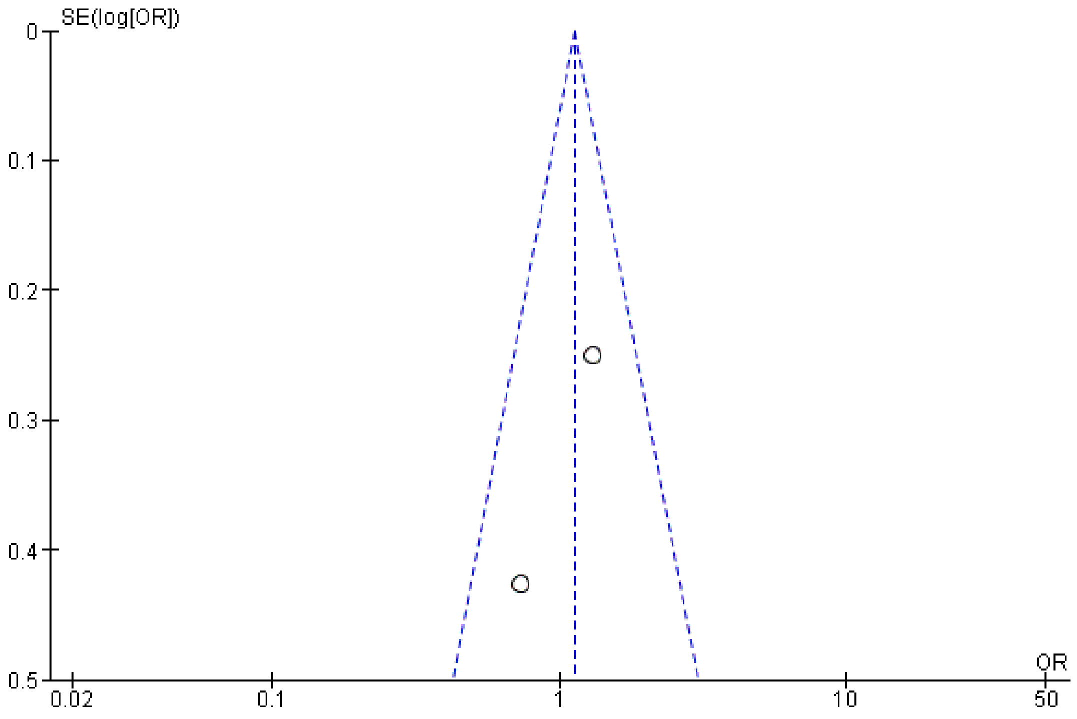

Figure S34. Morbidity funnel plot.

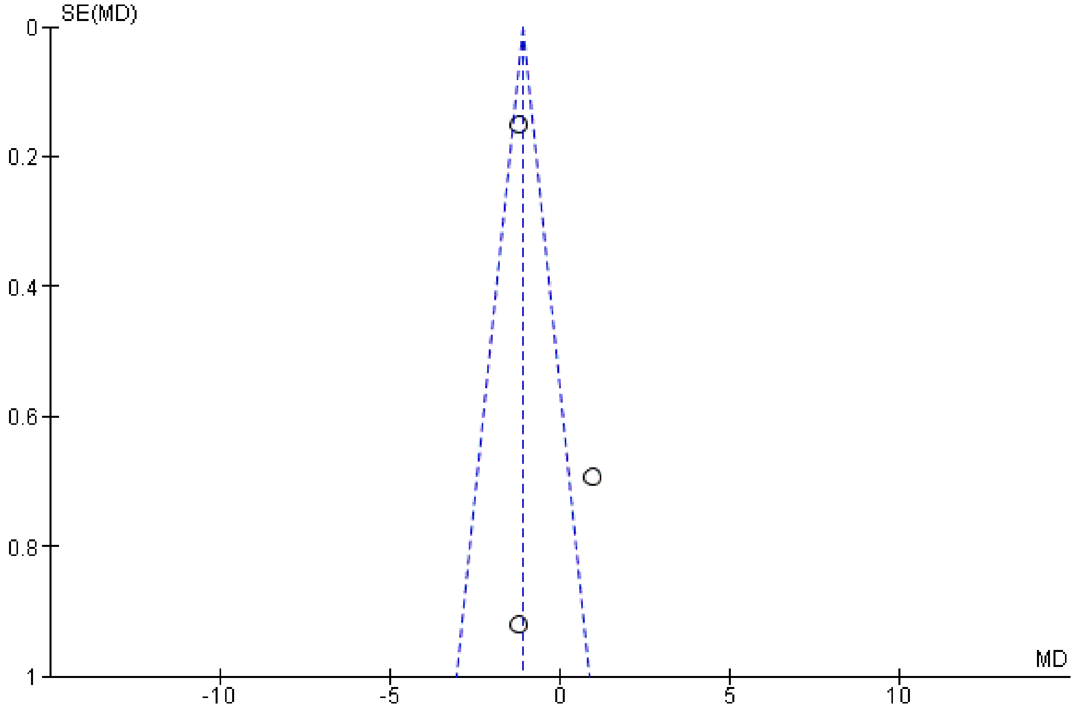

Figure S35. Length of hospital stay funnel plot.

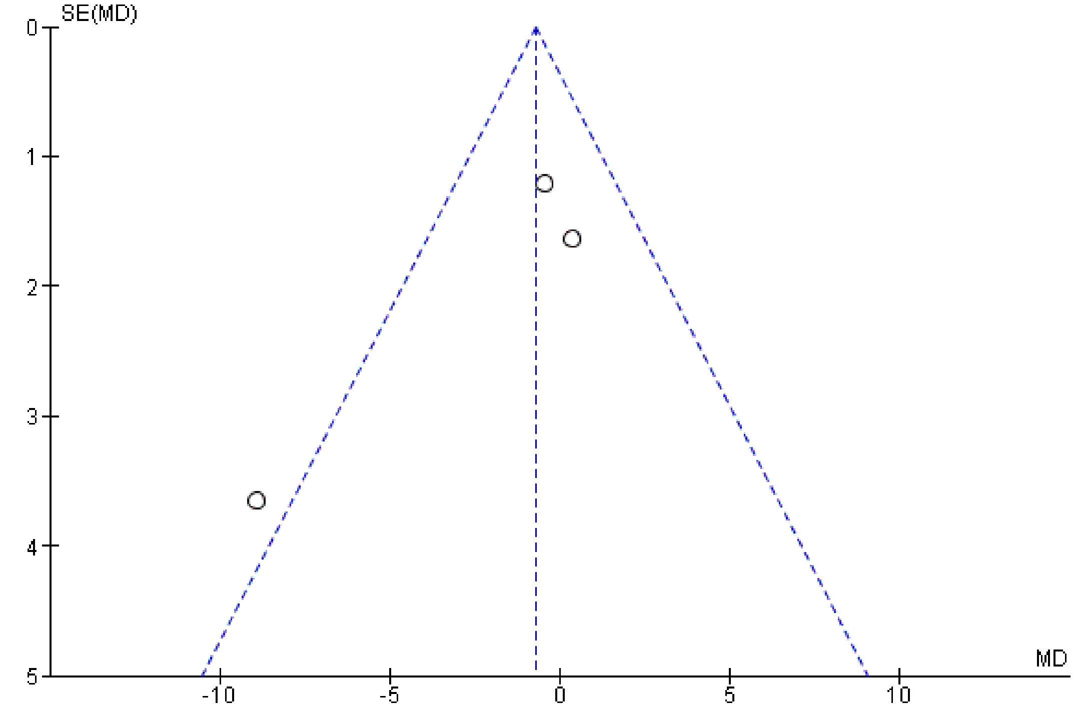

Figure S36. Lymph node harvest funnel plot.
